# Integration of Multi-Head Self-Attention and Convolution for Person Re-Identification

**DOI:** 10.3390/s22166293

**Published:** 2022-08-21

**Authors:** Yalei Zhou, Peng Liu, Yue Cui, Chunguang Liu, Wenli Duan

**Affiliations:** 1 School of Control and Computer Engineering, North China Electric Power University, Beijing 102206, China; 2 School of Electrical and Electronic Engineering, North China Electric Power University, Beijing 102206, China; 3 Yangzhong Intelligent Electric Research Center, North China Electric Power University, Yangzhong 212211, China

**Keywords:** person re-identification, surveillance, attention

## Abstract

Person re-identification is essential to intelligent video analytics, whose results affect downstream tasks such as behavior and event analysis. However, most existing models only consider the accuracy, rather than the computational complexity, which is also an aspect to consider in practical deployment. We note that self-attention is a powerful technique for representation learning. It can work with convolution to learn more discriminative feature representations for re-identification. We propose an improved multi-scale feature learning structure, DM-OSNet, with better performance than the original OSNet. Our DM-OSNet replaces the 9×9 convolutional stream in OSNet with multi-head self-attention. To maintain model efficiency, we use double-layer multi-head self-attention to reduce the computational complexity of the original multi-head self-attention. The computational complexity is reduced from the original $O({\left(H\times W\right)}^{2})$ to $O(H\times W\times G^{2})$. To further improve the model performance, we use SpCL to perform unsupervised pre-training on the large-scale unlabeled pedestrian dataset LUPerson. Finally, our DM-OSNet achieves an mAP of 87.36%, 78.26%, 72.96%, and 57.13% on the Market1501, DukeMTMC-reID, CUHK03, and MSMT17 datasets.

## 1. Introduction

People have been paying more attention to public safety in recent years. Driven by practical significance, the number of surveillance cameras in life and production is increasing. Researchers have extensively researched person re-identification (re-ID) under intelligent surveillance systems for public safety.

Some biometric features, such as face and gait, are more recognizable than visual appearance. Nevertheless, purely visual appearance is often more feasible due to the camera resolution and actual processing complexity in natural open-world surveillance systems.

Person re-ID aims to determine a person’s identity from a given probe image by calculating the similarity to the images in a given gallery (a set of candidates). Considering the lighting, posture, and perspective changes, the same person can look very different on different surveillance cameras. Moreover, different people will be very similar in some cases under the influence of dress and body type. Therefore, the re-ID, with larger intra-class and smaller inter-class variations, is more challenging than normal classification tasks.

Benefiting from the feature extraction capability of convolutional neural networks (CNNs), re-ID research has been greatly developed. Some supervised learning methods based on CNNs obtain good results [1,2,3]. Recently, the Transformer structure, which has seen great success in natural language processing (NLP), has attracted more and more scholars to study how to apply the Transformer structure in the 2D/3D structures of vision data [4,5]. One method combines the local information capture ability of convolution operation with the global relationship modeling ability of multi-head self-attention (MHSA). Inspired by this, we introduce our double-layer multi-head self-attention (DL-MHSA) based on OsNet’s multi-scale feature dynamic fusion [6], which enables the network to fuse features of different scales and global features. We call this network DM-OSNet.

In practical application scenarios, the large-scale unlabeled dataset LUPerson [7] has been proposed, which greatly facilitates relevant person re-ID unsupervised learning and domain adaptation learning [8,9]. Our improved network architecture will first perform unsupervised learning on the unlabeled dataset LUPerson instead of pre-training on ImageNet to improve model performance and generalization. To better fit the re-ID task, we use SpCL [10] as an unsupervised learning method.

Our contributions are summarized as follows:

1. We propose DL-MHSA based on MHSA, which reduces the computational complexity of MHSA with a small increase in the number of parameters. We replace the 9×9 convolutional flow of OSNet with our DL-MHSA, which not only improves the model performance, but also maintains the light weight of OSNet.
2. We use a large-scale unlabeled pedestrian dataset, LUPerson, instead of ImageNet for pre-training. Given that it is an unlabeled dataset, we sort and filter it with the help of the catastrophic forgetting score (CFS) [8] and, then, use SpCL to produce pseudo-labels for pre-training. The pre-trained model using this approach is tuned on the labeled dataset, and the model performance is further improved.
3. Our proposed method is comparable to most re-ID methods in the case of a lower number of parameters and FLOPs. More importantly, it is lightweight enough to facilitate deployments.

The other four sections of this paper are organized as follows. In “Related Work”, we briefly describe relevant work in the field of re-ID. In the the “Methods” section, we explain our proposed methodology. The experimental details, results, and analysis of these methods are available in the “Experiments and Analysis of Results” section. The ”Conclusion” section summarizes our study and provides an outlook for future research directions.

## 2. Related Work

### 2.1. Re-ID Based on Convolutional Neural Networks

Re-ID can be viewed as a kind of object classification problem. Some architectures for object categorization [11,12,13] are widely used for re-ID because of their excellent feature extraction capability. For example, in [14], the ID-discriminative embedding (IDE) uses ResNet50 as the backbone network, treating each ID as a separate class. It is worth noting that person re-ID differs from a standard classification task. First, most pedestrian images are rectangular regions cropped from the results obtained by pedestrian detection algorithms in different environments. Secondly, the pedestrian image itself is affected by the acquisition environment and the performance of the detection algorithm. It may have misalignment/occlusions problems. Finally, the re-ID tasks have common properties between classes and large intra-class differences. There is a significant amount of work addressing the re-ID task and designing some other methods that have advanced the development of pedestrian re-ID step by step. Ref. [2] decomposes the human body into parts and calculates the feature representation of different parts, then calculates the similarity of different parts separately. Ref. [3] used the pre-trained pose estimation model as a prior for the whole model to provide more accurate guidance for the alignment of the parts. However, more complex operations would increase the inference time. Some methods [3,15,16,17] use feature-level fusion to enhance local feature learning. Some works [18,19] try to use semantic segmentation to solve the background clutter problem. Compared with semantically meaningful body part partitioning using external cues, the method represented by PCB [20] uses a simple uniform partition strategy to directly horizontally divide region features, which is more flexible. However, refined part pooling (RPP) is needed to improve the performance. MGN [21] differs from previous global and local feature fusion approaches by proposing an end-to-end multi-branch architecture to obtain global and partial features. However, this architecture brings a large model size.

Influenced by multi-stream architectures such as Inception [22,23] and ResNeXt [24], some studies have also attempted to implement multi-stream aggregation in re-ID [6,25,26,27,28,29,30]. Ref. [25] draws on the ResNeXt structure and uses multiple factor modules in each block, which are considered to extract helpful semantic information for re-ID. Because pedestrian appearance differences are often subtle, more researchers [6,26,27,28,29,30] consider multi-scale feature learning meaningful for the re-ID task. These studies use different scales in each branch of the block to make the model adaptive in extracting features at different scales, thus enhancing the model’s ability to perceive different localities.

In recent years, attention-based mechanisms for feature representation have also received attention from researchers [31,32], which refers to making the network spontaneously find and learn information about regions of interest in images. The common strategy to use attention in re-ID is integrating a separate stream of regional attention into a deep convolutional re-ID model. Ref. [33] proposed joint pixel-level attention and channel attention. Ref. [34] proposed a comparative attention network, CAN, to simulate the human attention process. It repeatedly compares local parts of the human and pairs them. ABD-net [35] introduces the attentive branch to the ResNet50 architecture, focusing on channel aggregation and location awareness. SCAL [36] introduces additional supervised signals for attentional learning. By inserting a modified non-local attention module into ResNet, SONA [37] attempts to capture both non-local [38] and local correlations. Nevertheless, the non-local module is still computationally intensive. Ref. [39] introduces an attention module to make the model focus more on the foreground region instead of using an annotated mask.

### 2.2. Transformer in Visual Recognition

Transformer is a novel structure for extracting features using the self-attention mechanism [40]. Because of the powerful representation capabilities of Transformer and its success in NLP, some recent studies are trying to apply the Transformer architecture to the field of computer vision research. To use a pure Transformer on 2D images, ViT [4] simulates the structure of human utterances by dividing the input image into multiple patches projected into a vector. Some re-ID research work [41,42] uses the structure of the pure Transformer borrowed from the ViT backbone. Some works use Transformer in the CNN backbone to further aggregate features and information, such as AA-ResNet [43] and BoTNet [44]. There are also attempts in re-ID research to exploit the advantages of both the CNN and Transformer architectures. Ref. [45] uses Transformer on top of a CNN backbone to fuse multi-layer features of pedestrian images. Ref. [46] uses Transformer to obtain human part features in the CNN to discover the different human parts in occluded person re-ID.

## 3. Methods

**Figure 1:**
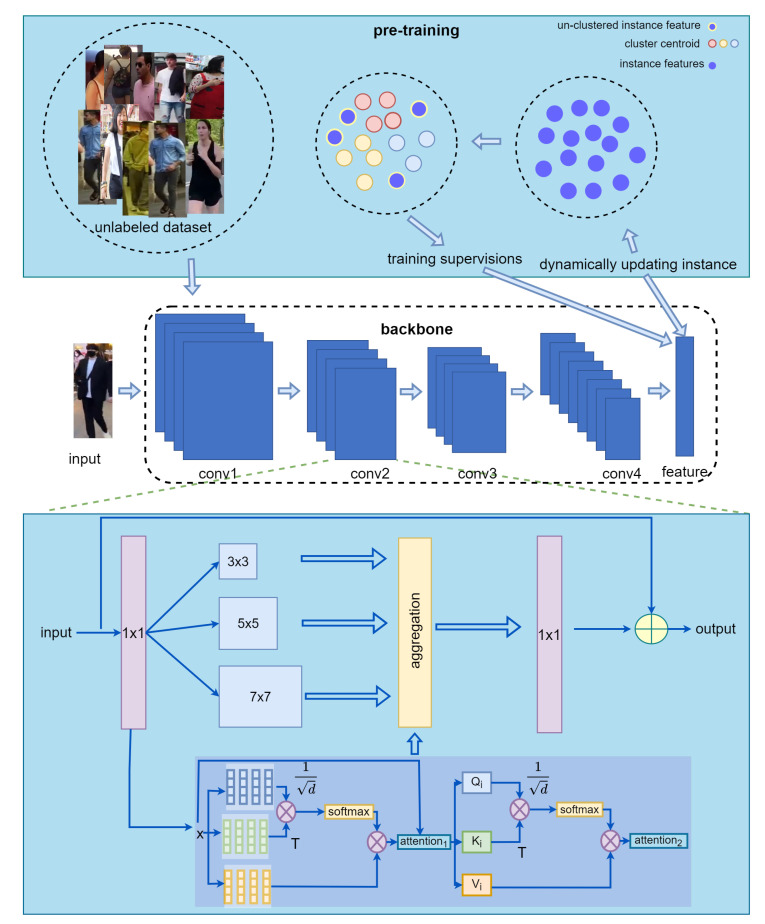
Methods to improve the ability of the lightweight model to extract discriminative features. One is to replace the convolution of the 9×9 receptive field in the original bottleneck of OSNet with our DL-MHSA. The other is to pre-train using a large-scale unlabeled pedestrian dataset.

In this section, we first introduce DL-MHSA. Then, we introduce the improved OSNet based on DL-MHSA. Finally, the pre-training method for the re-ID task using a large-scale unlabeled dataset is presented. A schematic diagram of these methods we propose is shown in Figure 1.

### 3.1. Double-Layer Multi-Head Self-Attention

MHSA is an important attention module in Transformer. MHSA first obtains the query Q, the keyword K, and the value V by applying three sets of projections to the input feature map $X\in R^{H\times W\times C}$, where H, W, and C are the height, width, and feature dimension of *X*. Then, we divide them into multiple parts, respectively. Each part of Q, K, and V and the subsequent processing represents a head. Q, K, and V are mapped from the original C dimension to the $d=\frac{C}{k}$ dimension in each head, where *k* represents the number of heads. We use $Q_{i}$, $K_{i}$, and $V_{i}$ to represent the required inputs in each head. The self-attention of each head can be expressed as

(1) $$head_{i}=\mathrm{SA}\left(Q_{i},K_{i},V_{i}\right)=\mathrm{softmax}\left(\frac{Q_{i}K_{i}^{T}}{\sqrt{d}}\right)V_{i}$$

**Figure 2:**
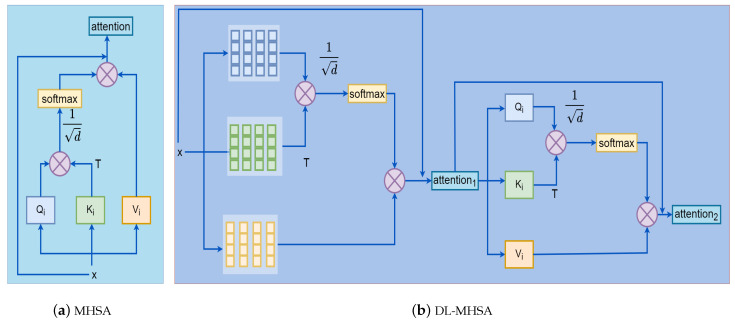
MHSA: structure of multi-head self-attention. DL-MHSA: structure of double-layer multi-head self-attention. T means matrix transpose.

Finally, the self-attention of multiple heads is stitched together to form a multi-head self-attention. The simplified structure of the MHSA is presented in Figure 2a, and note that we have omitted multiple heads here. The computational complexity of MHSA is influenced by the values of H and W, considering the matrix multiplication operation of the Equation (1). The time complexity of MHSA can be expressed as $O({\left(H\times W\right)}^{2})$.

To reduce the time and space complexity of MHSA, inspired by [47], we used a double-layer MHSA. We first divide the input feature map X with a G×G size grid in the first layer. By projecting the input X, we can obtain $Q_{i}$, $K_{i}$, and $V_{i}$ as

(2) $$Q_{i},K_{i},V_{i}\in R^{\left(\frac{H}{G}\times \frac{W}{G}\right)\times \left(G\times G\right)\times \frac{C}{k}}$$

Applying Equation (1), we can obtain:

(3) $$Q_{i}K_{i}^{T}\in R^{\left(\frac{H}{G}\times \frac{W}{G}\right)\times \left(\frac{H}{G}\times \frac{W}{G}\right)\times \frac{C}{k}}$$

(4) $$\mathrm{SA}\left(Q_{i},K_{i},V_{i}\right)\in R^{\left(\frac{H}{G}\times \frac{W}{G}\right)\times \left(G\times G\right)\times \frac{C}{k}}\to \mathrm{SA}\left(Q_{i},K_{i},V_{i}\right)\in R^{H\times W\times \frac{C}{k}}$$

We can obtain the feature mapping for the first layer when each head is computed:

(5) $${attention}_{1}=F\left({head}_{0},{head}_{1},\ldots ,{head}_{k}\right)+X$$

In Equation (5), F(.) stands for connecting operations on multiple heads’ inputs.

In the second layer, we map ${attention}_{1}$ to *Q*, *K*, and *V* as well, but only downsample *K* and *V* by using average pooling with the kernel size and stride of *G*, as

(6) $$Q_{i}\in R^{H\times W\times \frac{C}{k}},K_{i},V_{i}\in R^{\left(\frac{H}{G}\times \frac{W}{G}\right)\times \frac{C}{k}}$$

(7) $$Q_{i}K_{i}^{T}\in R^{\left(H\times W\right)\left(\frac{H}{G}\times \frac{W}{G}\right)}$$

(8) $$\mathrm{SA}\left(Q_{i},K_{i},V_{i}\right)\in R^{H\times W\times \frac{C}{k}}$$

We can obtain the final attention as

(9) $${attention}_{2}=F\left({head}_{0},{head}_{1},\ldots ,{head}_{k}\right)+{attention}_{1}$$

By the two-step operation, the computational complexity is decreased from the original $O({\left(H\times W\right)}^{2})$ to the present $O(H\times W\times G^{2})$ while still obtaining the self-attentiveness of the input feature map. We show the simplified structure of DL-MHSA in Figure 2. For clarity, we also show only one head.

### 3.2. OSNet Adds Self-Attention Transformer Stream

OSNet is an omni-scale feature learning network specifically designed for the re-ID task. It is achieved by designing a residual block consisting of multiple convolutional streams, each detecting features at a certain scale and, then, performing dynamic multi-scale feature fusion through a unified aggregation gate.

OSNet obtains different receptive field sizes by stacking multiple 3×3 convolutions in each convolutional stream. For example, two 3×3 convolutions are used to achieve a 5×5 convolution field. In the original OSNet, the 3×3, 5×5, 7×7, and 9×9 fields are aggregated in the residual block.

A part of the previous work added multiple branches on OSNet [48,49] to improve the model performance. Among these added branches, there must be a global branch, which shows the importance of global information for the re-ID task. The CNN-based model is more concerned with aggregating local information because it is not easy to obtain global information. In order to obtain global information, convolutional networks need to stack convolutional layers. Transformer-based models, in contrast, have the innate ability to acquire global information. A simple change in BoTNet [44], replacing the spatial 3 × 3 convolution layer with MHSA in the bottleneck blocks of ResNet, leads to an inspiring performance improvement. Inspired by this, we tried to integrate MHSA in the bottleneck of an OSNet so that it can gain the ability to integrate global information.

The main structure of OSNet’s residual bottleneck consists of a residual block of extended dimensionality and a unified aggregation gate. The residual block perceives multiscale features by multiple parallel convolutional streams of different receptive fields. Then, the unified aggregation gate aggregates features to capture a wide range of scales. We found that the convolutional streams in the residual block can be easily extended and replaced. Moreover, because there is a unified aggregation gate to provide a fine-grained fusion of input features, it is feasible to replace the convolutional stream that perceives the local receptive field with the self-attention stream that extracts global information about the features.

**Figure 3:**
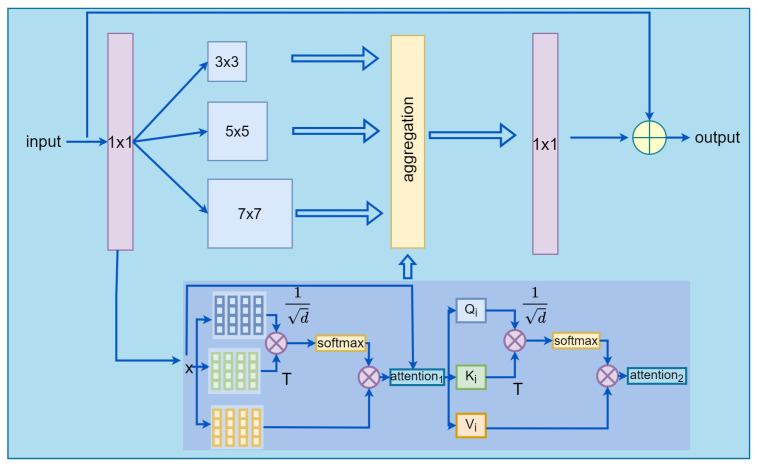
The bottleneck of DM-OSNet. The bottleneck aggregates the global self-attention stream and local feature stream.

The self-attention module uses a weighted averaging operation based on the input feature context compared to the convolution operation. The attention module can focus on different regions adaptively and capture more features. In this paper, we propose to replace the convolutional stream with a 9×9 receptive field by a double-layer MHSA to capture the long-range dependencies and global information, as shown in Figure 3. Suppose the input feature map to our improved bottleneck is *x* and the residual of the bottleneck is $\tilde{x}$. The acquisition of $\tilde{x}$ can be expressed as:

(10) $$\tilde{x}=\sum_{t=1}^{3}G\left(F^{t}\left(x\right)\right)⊙F^{t}\left(x\right)+G\left(H\left(x\right)\right)⊙H\left(x\right)$$

where $F^{t}$ represents the convolutional stream with a receptive field of (2t+1)×(2t+1). *G* represents the processing of features by the unified aggregation gate. ⊙ denotes the Hadamard product. H stands for our proposed double-layer multi-headed self-attention. Note that Equation (10) ignores the 1×1 convolution operation for clarity. Specific experiments can be found in the “Ablation Experiments Using DL-MHSA in Different Locations” sub-section under the “Experiments and Analysis of Results” section.

### 3.3. Large-Scale Unlabeled Dataset for Pre-Training

**Figure 4:**
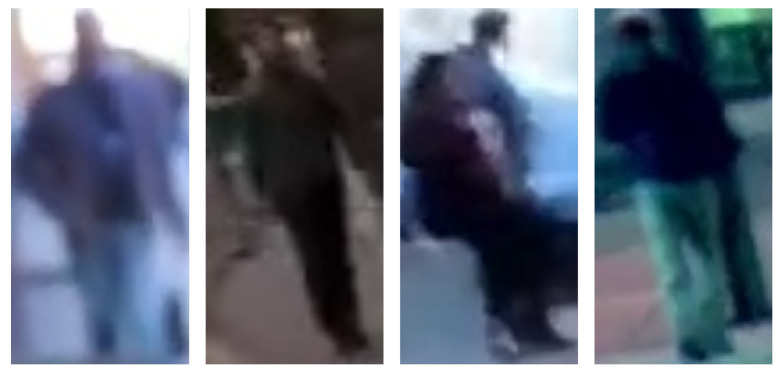
The LUPerson dataset contains low-quality pedestrian images.

**Figure 5:**
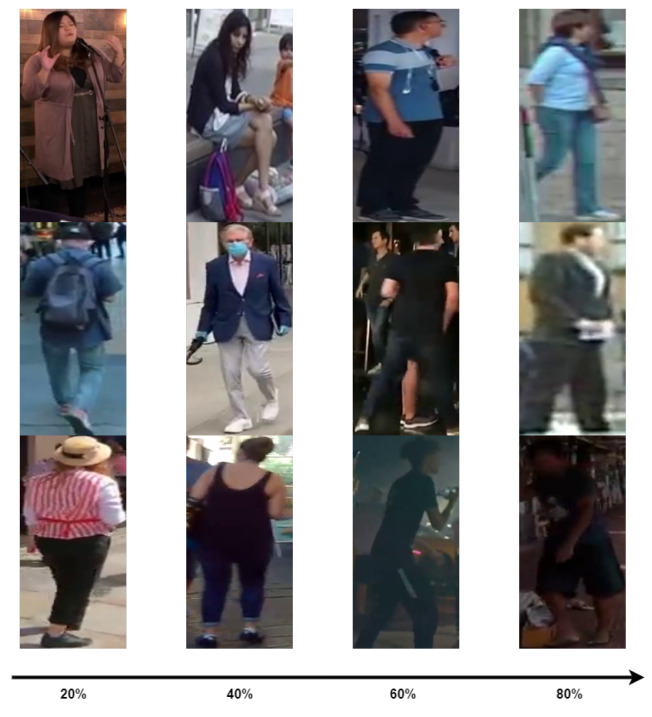
CFS sorts the LUPerson dataset. The percentages represent the position of these images in the sorted dataset.

LUPerson is the largest unlabeled dataset, with 4 million unlabeled pedestrian images and over 200,000 people. It was obtained by cropping pedestrians from videos. The previously proposed pedestrian re-ID networks are pre-trained on ImageNet, which is unsuitable for the person re-ID task because there is a huge domain gap between the dataset used for pedestrian re-ID and ImageNet. Our network uses LUPerson for the re-ID task pre-training. However, the original LUPerson dataset is too large, and there are some low-quality pedestrian pictures; see Figure 4. Moreover, because the data were collected from videos uploaded on video sites, although the scenes are rich, LUPerson has a domain gap with the dataset we often use. As pre-trained unlabeled data, LUPerosn is as close as possible to the downstream task, i.e., our supervised learning. To reduce the domain gap between the unlabeled and labeled datasets, we used the CFS to filter the LUPerson dataset. Figure 5 shows the outcome of the dataset sorted by the CFS in descending order. As can be seen, the lower the CFS ranking of the image, the worse the quality is. We chose the top 50% of the sorted images as our pre-trained unlabeled dataset according to the setting of [8]. The advantage of doing so is reducing the domain gap between the pre-trained and subsequently tuned datasets. Furthermore, this reduces the unlabeled dataset’s scale without weakening the pre-training operation’s performance. Moreover, using subsets saves pre-training time and cost.

We chose SpCL as the unsupervised method. SpCL is a pseudo-label-based method for unsupervised domain adaptation. We used LUPerson as the target domain data and did not use any labeled dataset as the source domain to pre-train our proposed network.

**Figure 6:**
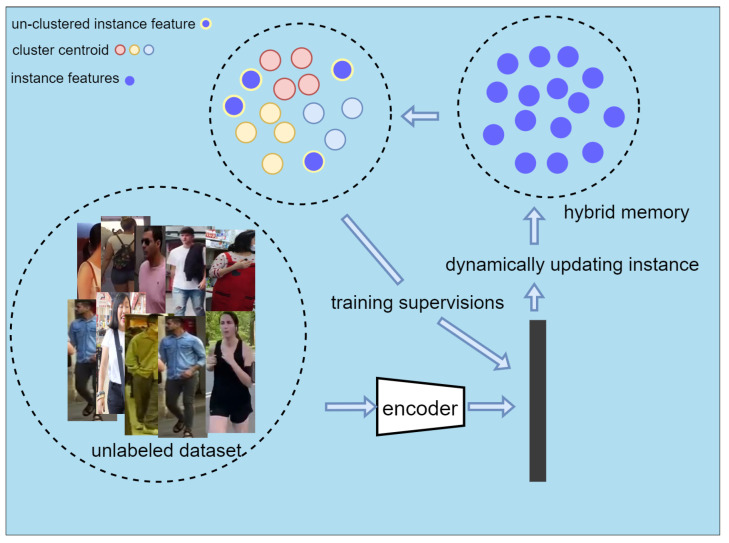
Schematic diagram of model pre-training. The training images are first encoded as instance features and stored in the memory. Subsequently, they are clustered and further processed into information that can be supervised for model training.

The model is pre-trained using SpCL for the unlabeled dataset, as shown in Figure 6. SpCL uses hybrid memory to store instance features and divides the features into cluster features and un-clustered features. The cluster centroid of each cluster is obtained by the mean of the features in each cluster. The cluster centroid and un-clustered features jointly supervise the training model by unified contrastive loss. Hybrid memory is initialized at the beginning of the iteration and dynamically updated in each iteration.

## 4. Experiments and Analysis of Results

### 4.1. Datasets and Evaluation Protocol

Datasets: We used four labeled datasets and one unlabeled dataset in our experiments. Among them, LUPerson is the unlabeled dataset used for pre-training. Market1501 [50], DukeMTMC-reID [51], CUHK03 [52], and MSMT17 [53] are labeled datasets used to show the efficacy of our proposed method experimentally.

LUPerson contains 4,180,243 images of pedestrians cropped from videos uploaded on video sites, with over 200k pedestrian appearances. All images are street videos from cities in different countries, and image names are coded by country, city, video, and frame rate.

Market1501, DukeMTMC-reID, CUHK03, and MSMT17 were collected from campuses. The first three are relatively similar regarding the number of pedestrian identities, with both having more than 1000 pedestrian identities. However, Market501 and DukeMTMC-reID have nearly three-times the total number of pedestrian pictures as CUHK03.

The original CUHk03 paper used a single-shot setting protocol, i.e., only one image per pedestrian in the gallery. We refer to the paper [54] and used a new training/testing protocol. The new protocol reorganizes the data in a format similar to Market1501, containing 767 and 700 pedestrian identities in the training and test sets, respectively. This makes it possible to have multiple images per pedestrian in the gallery, which is more suitable for real applications. Moreover, in the original protocol, the testing process had to be repeated 20 times. Using the new protocol avoids such repetition.

The number of pedestrian identities and pictures for MSMT17 exceeds Market1501 and CUHK03 by a large margin, with 126,441 pedestrian images and 4101 pedestrian identities, making it the largest labeled pedestrian dataset available. The details of each dataset are shown in Table 1.

Evaluation metrics: In our experiments, we used two evaluation metrics. One was the cumulative matching characteristics (CMCs). The CMC compares the similarity of all images in the query with all images in the gallery and sorts them separately. In the CMC, rank-n represents the hit rate of sorting the first n results that contain the correct label. The rank-n only considers the hit rate and does not provide a comprehensive assessment of the performance of the re-ID algorithm. The other was the mean average precision (mAP). Compared with rank-n, the mAP indicates the extent to which the correct images in the query results are ranked in front of the sorted list, which can better evaluate the algorithm.

### 4.2. Implementation Details

In our experiments, we set the size of all pedestrian images to 256×128. Our model was trained with a single NVIDIA 3090 GPU and pre-trained with 4 NVIDIA 3090 GPUs. Our implementation code refers to FastReID [55], and some experimental configurations followed the basic settings of this toolbox. For pre-training, we trained 30 epochs on the unlabeled dataset using SpCL. In the pre-training process, we set the learning rate to $3.5\times 10^{-4}$, the decay rate to 0.1, and the step size of the decay learning rate to 10 epochs. Since we used 4 GPUs for pre-training, our batch size per GPU was 16. In the subsequent tuning training, we trained the model for 120 epochs. During training, the base learning rate was $3.5\times 10^{-4}$ after 2000 iterations of warm-up. Then, the learning rate was maintained at $3.5\times 10^{-4}$ until 50 epochs. From 50 to 100 epochs, the learning rate decreased to $3.5\times 10^{-5}$. Finally, the learning rate decayed to $3.5\times 10^{-6}$ after 100 epochs until the end of training. In the training phase, the batch size was set to 64. Here, we empirically set the grid size in the first layer of DL-MHSA to 8×8. In the second layer of DL-MHSA, an average pooling with a kernel size and a step size of 8 was used.

### 4.3. Ablation Experiments Using DL-MHSA in Different Locations

OSNet stacks two bottlenecks in the second, third, and fourth convolutional layer (conv2, conv3, and conv4). Moreover, each bottleneck aggregates four convolutional streams. We replaced the convolutional stream with the maximum receptive field in the bottleneck with our proposed DL-MHSA. However, is this replacement effective? Which convolutional layer is it best to replace the bottleneck with DL-MHSA added? We performed an ablation study of the replacement design to answer these questions; see Table 2.

The baseline OSNet is represented in the experiments using [0,0,0] because DL-MHSA does not replace the 9×9 receptive fields in conv2, conv3, and conv4. To verify the effectiveness of the proposed DL-MHSA, we first replaced the 9×9 convolutional stream of conv2, conv3, and conv4, respectively. This substitution is denoted by [1,0,0], [0,1,0], and [0,0,1]. Then, we replaced the 9×9 convolutional streams in the two convolutional layers of the baseline with DL-MHSA. Finally, we replaced all 9×9 convolutional streams in all three convolutional layers with DL-MHSA.

Experimental results demonstrated that replacing the 9×9 convolutional stream in conv2, conv3, and conv4 brings performance gains to the model with DL-MHSA. However, this improvement is not obvious in higher layers of the model. Even on the MSMT17 dataset, the model’s performance significantly degrades when the 9×9 convolutional stream of conv4 is replaced. The performance degradation when using DL-MHSA at higher layers is due to the reduction in the feature mapping size. When the input image size is 256×128, the feature mapping size in conv2, conv3, and con4 is 64×32, 32×16, and 16×8. The higher the layer, the smaller the feature mapping size. MHSA itself is sensitive to the feature size, especially when we used DL-MHSA. We need to divide the feature mapping in the first layer and downsample the feature mapping in the second layer, which aggravates the size sensitivity of the network. The ablation experiments also demonstrated that using multiple DL-MHSA does not result in stacked performance gains for the model, especially when DL-MHSA is used in conv4. Considering the ablation experimental results, we subsequently used the [1,0,0] configuration for our DM-OSNet, i.e., instead of the 9×9 convolutional flow in conv2 for DL-MHSA.

### 4.4. Comparison of Different Pre-Training Methods

We used both the ResNet50 and OSNet models to verify that pre-training the model using the large-scale unlabeled pedestrian dataset LUPerson is more beneficial for the re-ID task than ImageNet. For ImageNet, we used supervised training so that the model initially learns the ability to classify images. For LUPerson, we used SpCL for unsupervised learning. Both models were pre-trained, followed by 120 epochs of supervised learning on three datasets (Market1501, DukeMTMC-reID, CUHK03, and MSMT17). Table 3 shows the results of the model performance evaluation.

From Table 3, we can see that after pre-training with LUPerosn, the mAP of ResNet50 on Market1501 was 1.28% higher than with ImageNet. Similarly, it was 2.6% higher on the CUHK03(D) dataset, 3.34% higher on DukeMTMC-reID, and 3.23% higher on MSMT17. When we switched to the original OSNet, the LUPerson pre-training improved by 1.26%, 3.12%, 1.19%, and 2.88% on the four datasets. These mAP precision improvements allowed us to demonstrate the effectiveness of LUPerson pre-training.

### 4.5. Comparison with Other Methods

**Figure 7:**
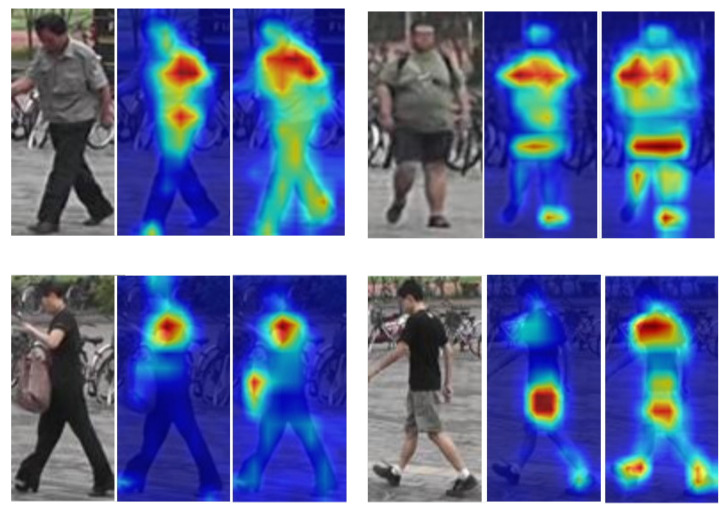
Activation maps of OSNet and DM-OSNet. From left to right are the input image, the activation map of OSNet, and the activation map of DM-OSNet.

Figure 7 demonstrates the two main advantages of DM-OSNet over OSNet through the attentional activation map. The first is that the model focuses on a larger area. See the first row of Figure 7. Second, the model focuses on more areas. See the second row of Figure 7. We believe this is due to the global information focus brought by DL-MHSA and the better model performance from pre-training on the large-scale unlabeled dataset.

**Figure 8:**
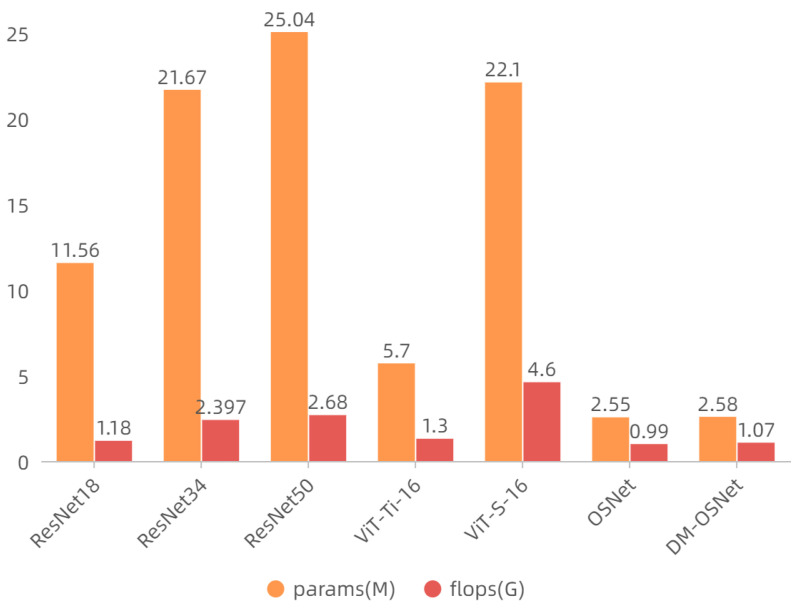
Parameter count and FLOP comparison. Params represent the number of model parameters. flops represents the number of floating point operations required by the model.

Table 4 shows the comparison of our proposed method with other methods. Our method has better or similar performance compared with other methods. It is worth noting that DM-OSNet does not add much to the OSNet baseline regarding the number of parameters and FLOPs. DM-OSNet has a smaller model size and computational requirements than other model backbones (see Figure 8).

**Figure 9:**
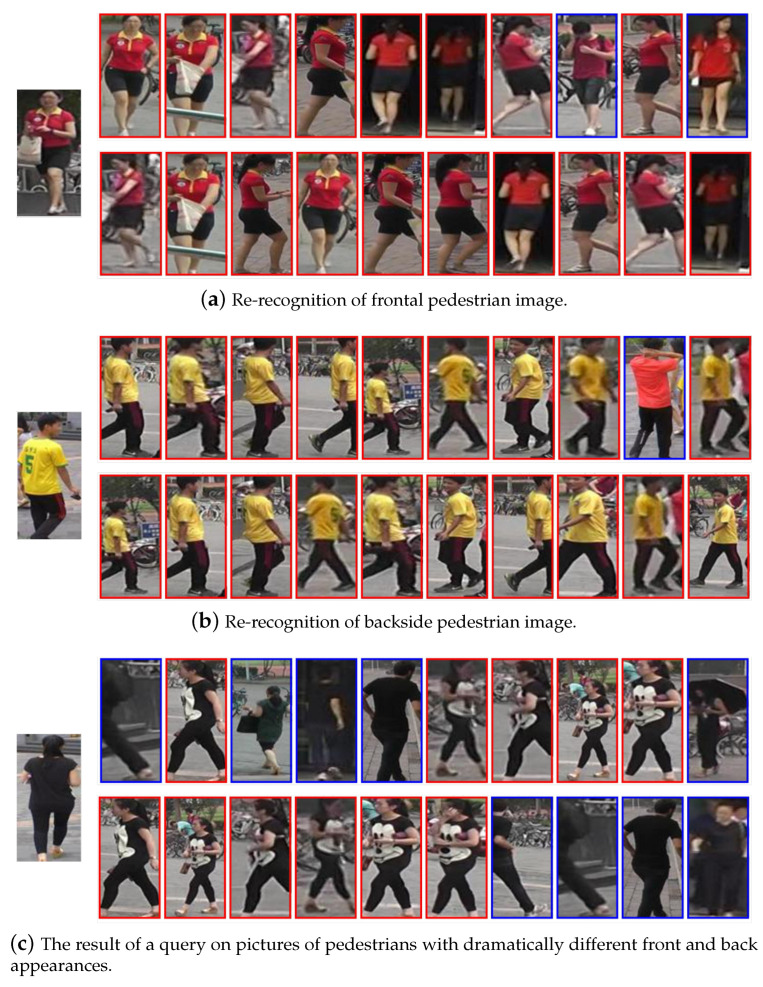
The rank-10 sorting of the query images. In each subfigure, the query image is shown on the left. The subfigure’s upper right and lower right areas display the query results for OSNet and DM-OSNet. Red and blue bounding boxes mark correct and incorrect matches, respectively.

Figure 9 shows the rank-10 visualization results of our proposed method on the Market1501 dataset. Our method is more robust than the baseline to different camera angles of pedestrians because of the inclusion of global information, such as the pedestrian front image query in Figure 9a and the pedestrian back image query in Figure 9b. They both obtained correct matches for pedestrian side shots. Because we used a feature extraction approach that fuses global and local information, our method still has good discriminative power when the pedestrian images are vastly different from different angles (The back of the pedestrian top in Figure 9c is solid black. However, the front is painted with a large pattern).

## 5. Conclusions

In this paper, we proposed DL-MHSA, which reduces the computational complexity of MHSA through a two-layer structure, but retains the global information focus capability of MHSA. We tried to apply this structure to the lightweight pedestrian re-ID network OSNet to improve the model performance while keeping the model as lightweight as possible. To this end, we proposed DM-OSNet, which was experimentally validated to perform better than the original baseline on four datasets, Market1501, DukeMTMC-reID, CUHK03, and MSMT17. We explored pre-training the pedestrian re-identification network to improve the model performance using the large-scale unlabeled dataset LUPerson and the domain-adaptive learning method SpCL. By sorting the LUPerson dataset by the CFS and selecting unlabeled images with high scores, we reduced the dataset size while optimizing the dataset quality. SpCL for unsupervised learning allowed our lightweight model to converge better and improved the performance. We plan to further explore the application of lightweight networks in realistic surveillance scenarios and enhance performance.

## Figures and Tables

**Table 1:** Statistics and comparison of the datasets. CUHK(D) represents the pedestrian bounding box obtained by the pedestrian detector. CUHK(L) represents the pedestrian bounding box obtained by manual labeling.

Datasets	Label	Images				ID			
		Total	Train	Query	Gallery	Total	Train	Query	Gallery
Market1501	labeled	32,668	12,936	3368	15,913	1501	751	750	751
DukeMTMC-reID	labeled	36,411	16,522	2228	17,661	1852	702	702	1110
CUHK03(D)	labeled	14,097	7365	1400	5332	1467	767	700	700
CUHK03(L)	labeled	14,096	7368	1400	5328	1467	767	700	700
MSMT17	labeled	126,441	30,248	11,659	82,161	4101	1041	3060	3060
LUPerson	unlabeled	4,180,243	-	-	-	46,260	-	-	-

**Table 2:** Ablation study on the replacement design in DM-OSNet. Bold indicates the best result on the dataset. [0,0,0] represents the original OSNet.

Method	Market1501		DukeMTMC-reID		CUHK03(D)		CUHK03(L)		MSMT17	
	mAP	rank-1	mAP	rank-1	mAP	rank-1	mAP	rank	mAP	rank-1
[0,0,0]	84.9	94.8	73.5	88.6	67.8	72.3	-	-	52.9	78.7
[1,0,0]	**86.76**	**95.26**	**76.87**	89.45	68.81	71.50	**71.56**	**74.00**	**55.96**	**80.25**
[0,1,0]	86.68	95.01	76.65	**89.54**	68.90	71.20	70.73	72.71	55.29	80.03
[0,0,1]	86.42	95.06	76.58	89.68	**69.09**	71.36	70.86	73.57	54.03	79.26
[1,1,0]	86.07	94.83	75.87	88.7	67.79	70.57	70.43	72.57	54.47	79.71
[1,0,1]	85.57	94.12	75.38	88.24	69.02	**71.93**	70.47	72.86	54.17	79.38
[0,1,1]	85.42	94.18	75.21	88.73	68.11	70.57	71.12	73.64	53.45	78.78
[1,1,1]	84.93	93.88	75.03	87.75	66.90	68.70	69.89	71.79	52.23	77.37

**Table 3:** Pre-training with the LUPerosn dataset and ImageNet for comparison.

Models	Pre-Training		Market1501		DukeMTMC-reID		CUHK03(D)		CUHK03(L)		MSMT17	
	Methods	Data	mAP	rank-1	mAP	rank-1	mAP	rank-1	mAP	rank-1	mAP	rank-1
ResNet50	Supervised	ImageNet	81.95	92.55	72.66	83.80	63.11	65.07	65.83	67.79	44.39	68.73
	SpCL	LUPerson	83.23	93.17	76.00	86.12	65.71	67.14	68.23	69.07	47.62	71.28
OSNet	Supervised	ImageNet	84.9	94.8	73.5	88.6	67.8	72.3	-	-	52.9	78.7
	SpCL	LUPerson	86.16	95.1	76.62	88.29	68.99	71.67	70.87	72.86	55.78	80.23

**Table 4:** The results of comparing the performance of our method with other methods on four generic datasets. The red and blue colored fonts in the table represent the best and second-best of these results, respectively.

Method	Market1501		DukeMTMC-reID		CUHK03(D)		CUHK03(L)		MSMT17	
	mAP	rank-1	mAP	rank-1	mAP	rank-1	mAP	rank-1	mAP	rank-1
PCB [20]	81.6	93.8	69.2	83.3	57.5	63.7	-	-	40.4	68.2
AANet [56]	83.4	93.9	74.3	87.7	-	-	-	-	-	-
DGNet [57]	86.0	94.8	74.8	86.6	-	-	-	-	52.3	77.2
OSNet [6]	84.9	94.8	73.5	88.6	67.8	72.3	-	-	52.9	78.7
Auto-ReID [58]	85.1	94.5	-	-	69.3	73.0	73.0	77.9	52.5	78.2
BDB [59]	86.7	95.3	76.0	89.0	69.3	72.8	71.7	73.6	-	-
IANet [60]	83.1	94.4	73.4	87.1	-	-	-	-	46.8	75.5
CAMA [61]	84.5	94.7	72.9	85.8	64.2	66.6	66.5	70.1	-	-
MHN [62]	85.0	95.1	77.2	89.1	65.4	71.7	72.4	77.2	-	-
SCAL [36]	89.3	95.8	79.6	89.0	68.6	71.1	72.3	74.8	-	-
MGN [21]	86.9	95.7	78.4	88.7	66.8	66.0	68.0	67.4	52.1	76.9
OSNet+DL-MHSA	86.76	95.26	76.87	89.45	68.81	71.50	71.56	74.00	55.96	80.25
OSNet++LUperson	86.16	95.1	76.62	88.29	68.99	71.57	70.87	72.86	55.78	80.23
OSNet+DL-MHSA+LUperson (DM-OSNet)	87.36	95.61	78.26	89.18	70.59	73.0	72.96	74.57	57.13	80.96

## Data Availability

Our data and code are available from this URL: https://github.com/yalei-zhou/DM-OSNet, accessed on 19 August 2022.
